# SIGNIFICANCE deep learning based platform to fight illicit trafficking of Cultural Heritage goods

**DOI:** 10.1038/s41598-024-65885-6

**Published:** 2024-07-02

**Authors:** Eva Savina Malinverni, Dante Abate, Antonia Agapiou, Francesco Di Stefano, Andrea Felicetti, Marina Paolanti, Roberto Pierdicca, Primo Zingaretti

**Affiliations:** 1 Dipartimento di Ingegneria Civile, Edile e dell’Architettura (DICEA), Università Politecnica delle Marche, Via Brecce Bianche 12, 60131 Ancona, Italy; 2 Eratosthenes Center of Excellence, Limassol, 3012 Cyprus; 3 The Cyprus Institute (CyI), Athalassa Campus, Nicosia, Cyprus; 4 VRAI - Vision Robotics and Artificial Intelligence Lab, Dipartimento di Ingegneria dell’Informazione (DII), Università Politecnica delle Marche, 60131 Ancona, Italy; 5 Department of Political Sciences, Communication and International Relations, University of Macerata, 62100 Macerata, Italy

**Keywords:** Energy science and technology, Engineering

## Abstract

The illicit traffic of cultural goods remains a persistent global challenge, despite the proliferation of comprehensive legislative frameworks developed to address and prevent cultural property crimes. Online platforms, especially social media and e-commerce, have facilitated illegal trade and pose significant challenges for law enforcement agencies. To address this issue, the European project SIGNIFICANCE was born, with the aim of combating illicit traffic of Cultural Heritage (CH) goods. This paper presents the outcomes of the project, introducing a user-friendly platform that employs Artificial Intelligence (AI) and Deep learning (DL) to prevent and combat illicit activities. The platform enables authorities to identify, track, and block illegal activities in the online domain, thereby aiding successful prosecutions of criminal networks. Moreover, it incorporates an ontology-based approach, providing comprehensive information on the cultural significance, provenance, and legal status of identified artefacts. This enables users to access valuable contextual information during the scraping and classification phases, facilitating informed decision-making and targeted actions. To accomplish these objectives, computationally intensive tasks are executed on the HPC CyClone infrastructure, optimizing computing resources, time, and cost efficiency. Notably, the infrastructure supports algorithm modelling and training, as well as web, dark web and social media scraping and data classification. Preliminary results indicate a 10–15% increase in the identification of illicit artifacts, demonstrating the platform’s effectiveness in enhancing law enforcement capabilities.

## Introduction

In the international scenario, a solid legislative framework has been developed to tackle cultural property crimes^1^. The year 2020 marked indeed the 50th anniversary of the UNESCO 1970 Convention on the Means of Prohibiting and Preventing the Illicit Import, Export and Transfer of Ownership of Cultural Property (The UNESCO 1970 convention. https://en.unesco.org/fighttrafficking/1970s), and the 25th anniversary of the UNIDROIT Convention on Stolen or Illegally Exported Cultural Objects (Unidroit convention on stolen or illegally exported cultural objects. https://www.unidroit.org/instruments/ cultural-property/1995-convention). However, the illicit trade of cultural goods continues to damage the heritage worldwide and fosters criminal behaviour through its illegal profits^2^. The consequences of these crimes were first recognized in 1954 with ‘The Hague’ Convention. Afterwards, a large number of international instruments have been adopted, such as the UN Convention against Transnational Organized Crime^3^, the UNESCO Convention on the Protection of the Underwater Cultural Heritage^4^; and the Council of Europe Convention on Offences relating to Cultural Property^5^. Reliable statistical descriptions of the material and monetary volume of the illicit trade in antiquities don’t exist in detail. The World Customs Organization (WCO) “...estimates the size and profitability of black markets in looted, stolen or smuggled works of art [..] is worth billions of US dollars”. Recently, this criminal behaviour has been encouraged by the COVID-19 pandemic, which has affected the surveillance of archaeological sites and museums^6^. In countries experiencing conflict or post-conflict, the pandemic has exacerbated an already fragile security situation. The scale of this profitable crime is increasing globally, even thanks to the dissemination of online illicit conventions^7^. Social media platforms, for example, host groups dedicated to illegal archaeological excavations and illicit trade of cultural goods. Looters have the freedom to connect online with potential buyers around the world. The scenario is worsened by the intrinsic richness of cultural object classes that are illicitly traded: different periods, cultures, materials, sizes, regions, and associated values. Trafficking of cultural artefacts is more extensive now than ever before. The low-risk, high-profit aspect of art theft, political and security breakdowns in countries that came to encompass territories of ancient civilizations, provide ideal conditions for these illegal activities^8^. Most EU countries do not have the means to review all offers of a suspicious nature nor to efficiently monitor threats on online channels. Monitoring crimes against Cultural Property on internet is indeed a matter of scale. Analyzing the activities of hundreds of thousands of users poses several challenges due to the sheer volume and diversity of items offered or discussed and the limited reaction time available. Since the number of antiquities traded will likely continue, and looted antiquities may disappear for years before reappearing on the market. Thus, the need for continuous monitoring is mandatory. As highlighted in many reports, policies, and initiatives (i.e., EUROPOL - Serious and Organised Crime Threat Assessment; EU Policy Cycle on Serious and Organised Crime; EMPACT - European Multidisciplinary Platform against Criminal Threats), and recently, in the EU Security Union Strategy, “Trafficking in cultural goods has (..) become one of the most lucrative criminal activities, and it is on the rise. Steps indeed should be explored to improve the online and offline traceability of cultural goods in the internal market [..] providing active support to law enforcement and academic communities”.

In response to the aforementioned challenges, the European project “Stop Illicit heritaGe traffickiNg with artiFICiAl iNtelligenCE” (SIGNIFICANCE) was born (http://www.significance-project.eu) ^9^. It aims to enhance the responsiveness and effectiveness of public authorities and law enforcement agencies in combating the online illicit trafficking of cultural goods. In this paper, we present the results of the SIGNIFICANCE project, building upon previous preliminary works in the field of combating illicit trafficking of cultural goods using Artificial Intelligence (AI) and Deep Learning (DL) techniques^9,10^. Additionally, we introduce a novel AI-based platform that offers interactive visualization of the results obtained during the web and social media scraping and DL phases. The SIGNIFICANCE platform enables competent authorities to identify, track, and block illegal activities online, thus ensuring successful prosecution and revealing criminal networks. By exploiting AI, in particular, DL algorithms, the platform interfaces with the web, social media, and the dark web, for the automatic identification of genuine and counterfeit artefacts and potentially links them to criminal networks. Through AI-based image and text analysis techniques SIGNIFICANCE identifies suspicious activities and reports them to relevant authorities for swift response and a better understanding of the scope of online antiquities trafficking networks^10^. Despite their potential, in fact, AI and DL are rarely adopted to address the illicit trafficking of cultural goods, struggling to fully exploit their new possibilities. One key contribution of this paper is the development of a user-friendly platform that allows authorities and law enforcement agencies to visualize and interact with the results obtained during the web and social media scraping and classification processes. Furthermore, the platform incorporates an ontology-based approach, providing comprehensive information about the cultural significance, provenance, and legal status of identified artefacts. This allows users to access a wealth of knowledge and contextual information related to the items encountered during the scraping and classification phases, supporting informed decision-making and targeted actions. The project aims to enhance intelligence investigations, including cross-border cooperation among EU and non-EU member states, with a target of annually increasing the identified quantity of artefacts sold or advertised online by an average of 10% to 15% nationally. The platform developed brings new levels of efficiency and reliability to detection and investigation methodologies, tailored to the needs of different control authorities.

Recent developments in artificial intelligence (AI) have been particularly influential in the field of security. The European Union (EU) is concentrating its efforts on creating AI solutions that adhere to three principal standards: legality, ethics, and sturdiness. These standards are in line with the EU’s guidelines for trustworthy AI (https://digital-strategy.ec.europa.eu/en/library/ethics-guidelines-trustworthy-ai) and are in anticipation of the proposed EU AI Act.

(https://www.europarl.europa.eu/news/en/headlines/society/20230601STO93804/eu-ai-act-first-regulation-on-artificial-intelligence), ensuring that international laws are respected, especially regarding ethical considerations for sensitive data. Research has shown that AI methods which rely on data can enhance consumer protection by helping to automate the analysis of legal documents^11^. AI also contributes to safeguarding children using media devices like smartphones and tablets^12^ , preventing the accidental spread of private information online, and protecting the interests of people who perform tasks for online platforms, commonly known as crowdworkers^13^. In the area of protecting cultural property, the project SIGNIFICANCE was pioneering the use of AI to combat the illegal trade of cultural artifacts. Several initiatives have then followed such as ENIGMA (https://eu-enigma.eu/), AURORA (https://www.aurora-euproject.eu/), and RITHMS (https://rithms.eu/), which have continued to advance the use of AI in this vital domain.

The SIGNIFICANCE methodology is strict adherence to ethical guidelines and the legal regulations are maintained. The data collection process respected privacy and data protection principles, ensuring that personal information was anonymized and handled securely. The study is also conducted in collaboration with relevant authorities and complied with applicable laws and regulations regarding data acquisition and handling. By following this methodology and using the visualization platform, we aim to effectively detect and prevent the illicit movement of cultural heritage (CH) goods through a combination of DL, ontology-based classification, real-time monitoring, and interactive data visualization within an ethical framework^14,15^.

In summary, the main contributions of this paper, which aims to fulfill the gap in illicit traffic of cultural goods, are: (i) a novel user-friendly platform that offers interactive visualization of results obtained from web scraping, social media analysis, and DL processes; (ii) the importance of the use of AI and DL in combating this criminal activity more effectively; (iii) an ontology-based approach for data annotation; (iv) the use of an infrastructure that optimizes computing time, resource allocation, and cost efficiency.

The remaining sections of this paper are organized as follows: “Related work” provides an overview of relevant related works in the field of AI for image classification in cultural heritage. Section “Methods” presents the methodology employed in this study. The experimental setup and obtained results are discussed in “Results and discussions”. In “Results and discussions”, various analyses enabled by the system are presented. Finally, “Conclusions and future works” concludes the paper and outlines potential avenues for future research.

## Related work

This section presents an overview of significant research conducted in image classification within the cultural heritage domain, including multimodal classification approaches. Developing an effective image classification model for CH data offers substantial benefits in enhancing public understanding of diverse cultures. Extensive efforts have been made in this area, which can be broadly categorized into three main methods: handcrafted feature-based approaches, machine learning techniques, and deep learning methods. Image processing techniques have diverse applications in (i) Cultural Heritage (Hurtut et al.^16^, Makridis and Daras ^17^, Can et al. ^18^, Hu et al. ^19^); and (ii) architectural heritage (Shalunts et al. ^20^, Mathias et al. ^21^, Chu and Tsai ^22^, Goel et al. ^23^, Oses and Dornaika ^24^, Zhang et al. ^25^, Xu et al. ^26^, Amato et al. ^27^). These works highlight the versatility and significance of image processing in preserving and analyzing both cultural artifacts and architectural structures.

Convolutional Neural Networks (CNNs) have demonstrated exceptional performance in various computer vision tasks, as shown by Chen et al.^28^, Pandit et al.^29^, and Li et al.^30^. In Cultural Heritage image classification, Ćosović and Janković^31^ eused a CNN-based neural network to predict categories, showcasing CNNs’ superior capabilities. Kulkarni et al.^32^ proposed a transfer learning approach for monument classification. Recently, Lamas et al. explored deep learning techniques, specifically CNNs, for classifying architectural heritage images ^33^. Fan et al. ^34^, introduced a multimodal image classification model with late fusion (MICMLF), incorporating modal representation layers, a multimodal attention mechanism, hierarchical fusion, and a late fusion scheme.

In the context of illicit traffic of CH goods, Winterbottom et al.^35^ developed a machine learning-based framework for instance classification of large archaeological image datasets. They focused on several classes in the Durham Oriental Museum, collecting a dataset comprising over 24,502 images of 4332 unique object instances.

Our paper extends the aforementioned study in several significant ways, enhancing the understanding and capabilities in detecting and preventing the illicit movement of CH goods. While the previous study focuses on detecting known artifacts in archaeological image datasets, our paper broadens the scope by addressing the identification and prevention of illicit traffic of CH goods. We achieve this by scraping data from various sources, including the web, social media platforms, and the dark web. By incorporating these additional data sources, we gain a comprehensive view of the online landscape where illicit activities may occur. In addition, in contrast to the previous study based on machine learning instance classification approach, our paper employs a DL approach combined with an ontology-based framework. Moreover, our paper emphasizes the practical application of the research in a real-world context. This enables a deeper understanding of the patterns, trends, and networks associated with the illicit movement of cultural heritage goods. In Table 1, we present a comprehensive comparison of existing research in the field of image processing techniques and CNN approaches in relation to our developed platform. Our aim is to highlight how our approach builds on and goes beyond the existing literature, particularly in the context of using AI and deep learning for CH protection and preservation. The comparative analysis serves as a basis for understanding the novel contributions and enhanced capabilities of our work, particularly in addressing the challenges of illicit trafficking of CH goods.

**Table 1:** Comparative summary of existing work related to the developed platform, highlighting the main aspects, similarities, differences, strengths and weaknesses in relation to our research.

Category	Topic	SIGNIFICANCE added value
Image processing techniques	Geometric information & Stroke analysis^16^	Limited relevance to the fight against illicit trafficking of cultural goods. The SIGNIFICANCE project goes beyond this by integrating AI for proactive detection and prevention
	Color and Texture analysis^17^	Basic techniques, but not directly applicable to illegal traffic monitoring. SIGNIFICANCE uses advanced AI techniques for comprehensive analysis
	Shape representations^18,19^	Insightful in shape representation, less focused on illicit traffic. SIGNIFICANCE integrates these concepts with DL for improved artefact identification
	Architectural style & Local features^20–26^	Focuses on style classification, not illicit traffic monitoring. SIGNIFICANCE uses these techniques to identify and track cultural heritage objects
	Landmark recognition^27^	Techniques that do not target the illicit trafficking of cultural goods. SIGNIFICANCE enhances this with AI-driven contextual analysis for protective measures
CNNs approaches	Various vision tasks^28–30^	General performance, not specifically related to SIGNIFICANCE. The project uses advanced CNNs for targeted identification and prevention of illegal activities
	Cultural heritage classification^31–33,36^	Improved ability to identify illicit trafficking of cultural heritage. SIGNIFICANCE takes this further with integrated AI for more effective monitoring and prevention
	Multimodal and illicit traffic classification^34,35^	Advanced analytics to help combat illegal activities; directly addresses the challenge of illicit traffic. SIGNIFICANCE integrates multimodal data analysis for comprehensive cultural heritage protection

## Methods

In this section, we delve into the methodologies employed in our project, offering a comprehensive overview of the systematic steps comprising our workflow.

- Data extraction and storage. SIGNIFICANCE comprises a comprehensive data collection approach. This involved scraping data from various online sources, including from various selling portals on the web (Catawiki, eBay, Trocadero), social (Instagram and Facebook), and dark web. We use custom-built web crawlers and data extraction techniques to retrieve relevant information related to CH artifacts, such as images, descriptions, seller profiles, and transaction details.
- CH database and data annotation. Based on the collected data, we constructed a dataset specifically tailored for the task of detecting illicit traffic. The dataset consisted of a wide range of CH artifacts, encompassing different types, time periods, and regions. To enhance the classification accuracy and provide additional contextual information, we integrated an ontology-based approach into our framework. This ontology facilitated a more nuanced classification process by incorporating domain-specific knowledge and relationships between artifact attributes.
- AI module. Based on the trained DL model and ontology, we developed a classification approach for CH goods. Each artifact was represented by multiple images and associated metadata, providing a rich and diverse collection of samples for analysis. To analyze and classify the collected artifacts, we employed a DL framework. Specifically, we use a CNN architecture due to its effectiveness in image classification tasks. The CNN was trained on the collected dataset using a large-scale supervised learning approach.
- SIGNIFICANCE platform. To facilitate the interpretation and analysis of the results obtained from the detection and prevention framework, we developed a user-friendly visualization platform.

**Figure 1:**
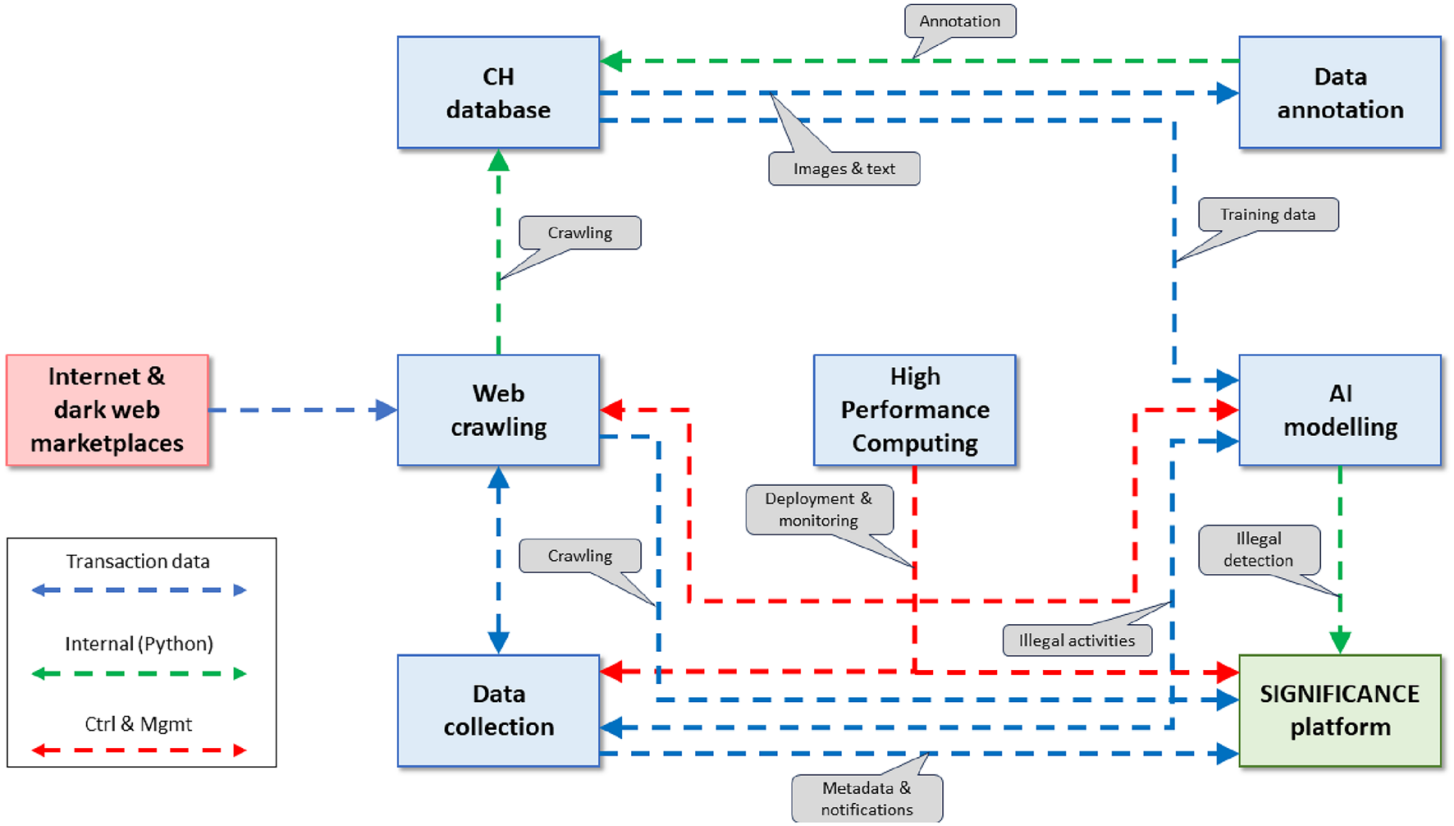
SIGNIFICANCE architecture detailing the interfaces between the different components as well as the data exchanged and functions between them.

A graphical representation of these steps is given in Fig. 1 and each of them has been described in more detail in the following subsections. The source code, experimental settings, and supplementary materials are available in our GitHub repository https://github.com/vrai-group/significance.

### Data extraction and storage

This step represents the first phase of the entire process: a scraping algorithm from multiple sources. A crawler is implemented for each source. By a text-based query (as in a search engine), a list of products (on selling portals) or posts (on social media) satisfying the query is obtained. Crawlers can be executed at any time, and each time they are executed, the timestamp of the last execution is kept. In this way, by adding the condition “publication date greater last execution date” to the query, it is possible to increase the database with new items while avoiding adding duplicates. For each item, downloaded images are stored in a common folder, while textual information and image references are stored in a JSON file in a MongoDB (https://www.mongodb.com/). The Instagram crawler makes use of the Instaloader library (https://github.com/instaloader/instaloader) to connect and download posts from Instagram. The data is collected by searching by hashtags and downloading only information relevant to the target such as: the post image, post description, hashtags, user information, etc. The eBay crawler makes use of the libraries *beautifulsoup4*, *selenium*, and *requests*. The data are collected by retrieving the info visible on the web page associated with the individual product from the HTML content. The information stored are: price, seller, payment method, etc.

### CH database and data annotation

#### Dataset

**Figure 2:**
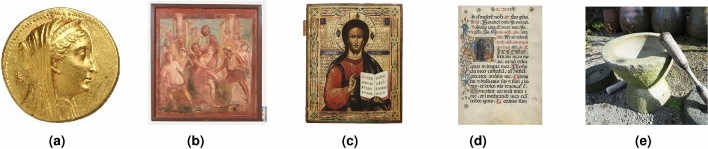
Samples of SIGNIFICANCE dataset. (**a**) Coins. (**b**) Frescoes. (**c**) Icons. (**d**) manuscripts. (**e**) Other.

The following image datasets were collected and labelled as previously defined (Fig. 2). For each artwork, the images were labelled according to a feature/field of each work of art (e.g., location, periods, material, etc.) and different classes belong to each field (Table 2).

**Table 2:** Images labelling according to a feature/field of each work of art (e.g., location, periods, material, etc.).

Coins	Frescoes	Icons	Manuscripts
Material: gold, silver, other	Location: cyprus, italy, other	Location: cyprus, other	Location: france, italy, other
	Period: byzantine, other		Language: latin, other
			Description: figure, text, figure and text

#### Ontology-based data annotation

**Figure 3:**
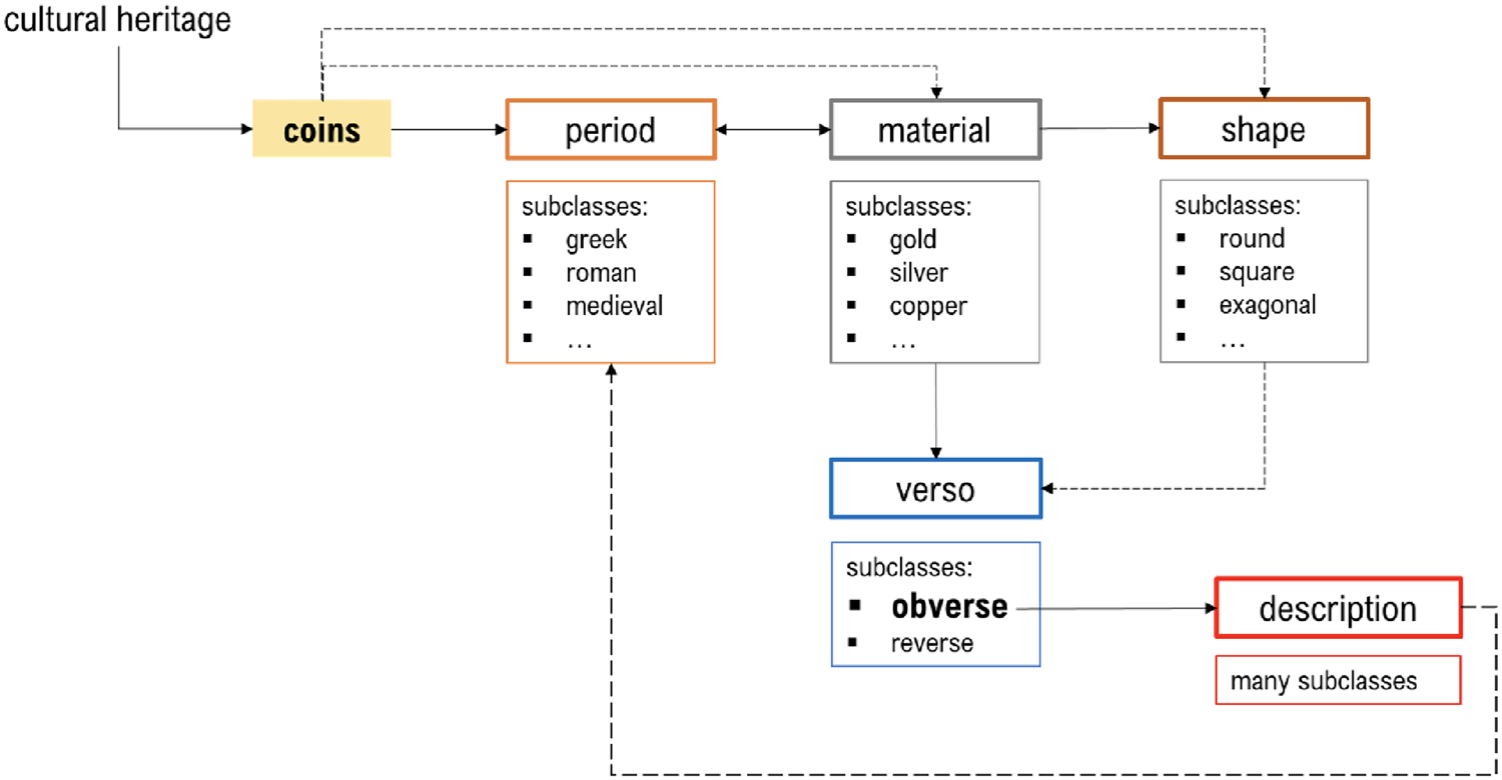
Example of ontological scheme for coins.

Given the diverse nature of online content, encompassing text, audio, video, and images, a knowledge-based modeling approach is indispensable^37,38^. In order to create a robust pipeline applicable to a broad spectrum of artifacts within the SIGNIFICANCE project, we advocate for an ontology-based image classification and annotation approach. To facilitate the implementation of the SIGNIFICANCE Project, we have selected the Web Ontology Language (OWL) and Protégé, an open-source ontology editor, to construct intelligent systems and knowledge-based solutions. The user-friendly WebProtégé environment enables collaborative ontology development and customization, streamlining the process. The ontology is constructed to represent the semantic hierarchy of image categories derived from training images. Notably, we have identified specific classes crucial for recognition, including coins, frescoes, icons, and manuscripts. Each artwork’s images are labeled according to various features/fields such as location, periods, material, etc., with different classes corresponding to each field. For the CH domain, existing ontologies are already available, and for the implementation of the SIGNIFICANCE project, the comprehensive and up-to-date ArCo (http://wit.istc.cnr.it/arco) ontology has been chosen. Taking coins as an illustrative example, the ontological scheme has been developed based on aspects determining relevant information for factors like period, material, shape, verso, and verso description (Fig. 3). Following the definition and construction of the ontology, the training phase of visual-feature classifiers is executed based on taxonomic relationships between classes. An unchangeable ID numerical code, reflecting the classification assigned by INTERPOL, has been allocated to each cultural heritage object. This code, incorporating a combination of numbers and words, serves as a unique identifier for queries and complements the ontological annotation containing all recognized information for each object.

### AI module

The AI module employed in the SIGNIFICANCE project involves the ontology-based classification approach (“CH database and data annotation”) for processing and categorizing images of artworks. This approach aims to extract valuable information about the depicted artwork through a two-stage process. Firstly the artwork depicted in the image is classified among 5 classes: coins, frescoes, icons, icons, manuscripts, and others. The use of the class others was necessary to classify images not belonging to the 4 already defined. The decision to use the class “others” is twofold. First it will address a hierarchical classification where images classified as “others” will be subject to further classification into more specific categories as more data becomes available^39,40^. Second, the images classified as “others” will be used as a benchmark to assess whether the defined classes are well-chosen and mutually exclusive. After the image has been classified at the first stage, it goes to the stage classification models (specific to the type of artwork) to extract specific features (e.g., location, period, etc.). In order to classify images, we use the neural network VGG16^41^, since it is a fast and reliable network, that has proven to be capable to solve many classification tasks over the years. The classification task comprises two phases: Artworks classification and Artworks features classification. The classes identified by domain experts as the most important to recognize are: coins, frescoes, icons, manuscripts and others. The approach for the class identification was based on a user requirements questionnaire which was distributed among Law Enforcement Agencies, Public Authorities and heritage experts. The survey gathered more than 40 responses. The proposed classes reflected the taxonomy of the ICOM Object ID, a standardized reference to document and describe collections of archaeological, cultural, and artistic objects (https://icom.museum/en/resources/standards-guidelines/objectid).

### SIGNIFICANCE platform

The SIGNIFICANCE platform provided interactive visualizations and dashboards that showcased the detected illicit activities, patterns, and insights derived from the collected data. Users, including law enforcement agencies and relevant authorities, could explore and analyze the data, gaining valuable insights into the scope and dynamics of the illicit trade of cultural heritage goods. The visualization platform enabled informed decision-making, proactive measures, and targeted actions against illicit traffickers. For the achievement of the project goals, several tasks, which demand serious computational resources, are run on the CyClone HPC Infrastructure of The Cyprus Institute specifically configured for AI processing activities. This approach allows to optimize the procedures in terms of computing time, resources allocated and cost-efficiency. The two tasks which mainly benefit from the use of the HPC infrastructure are: Web Scraping and Data Annotation, Algorithm Modelling and Training.

## Results and discussions

**Figure 4:**
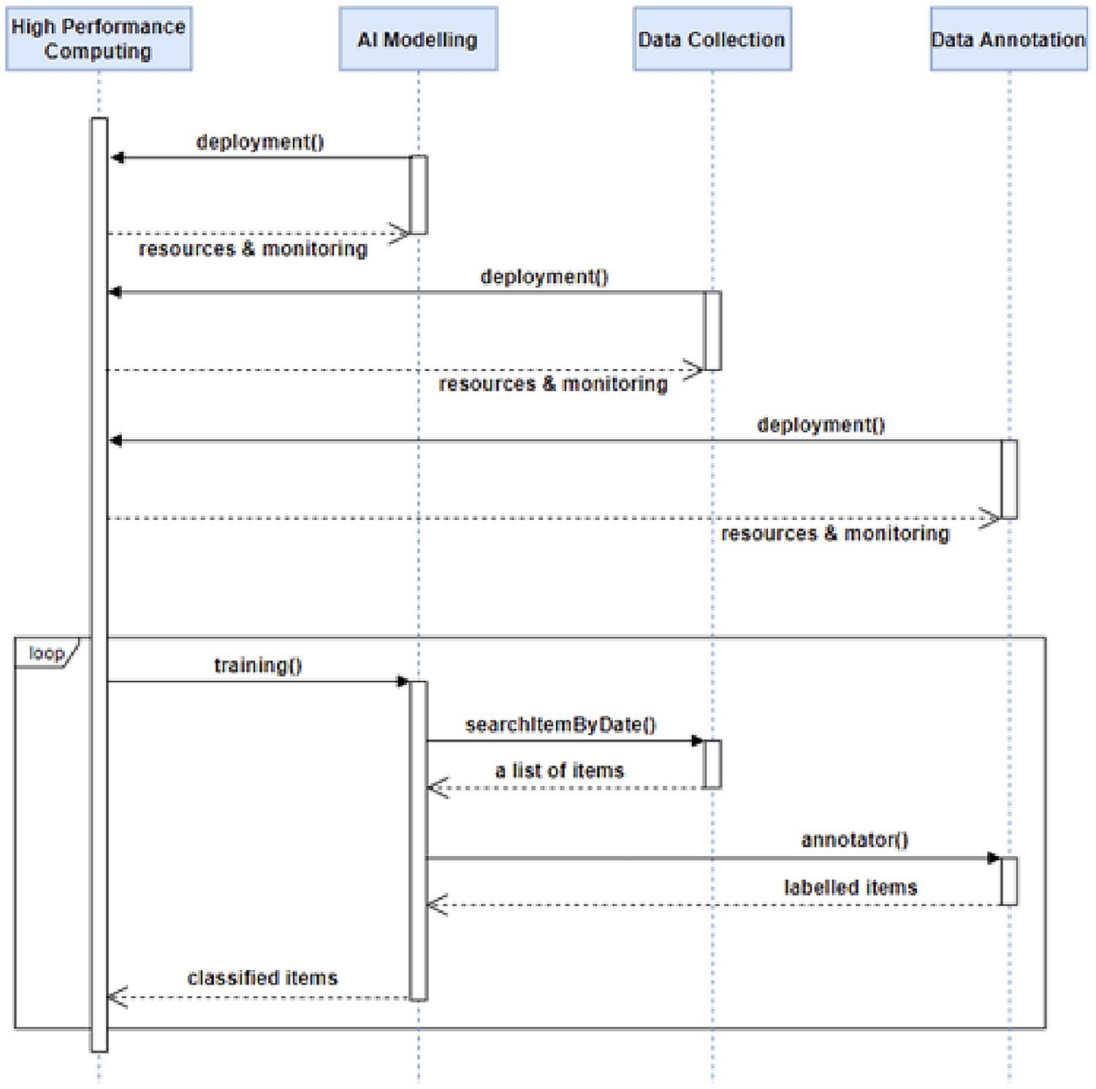
The sequence diagram of development of AI models.

**Figure 5:**
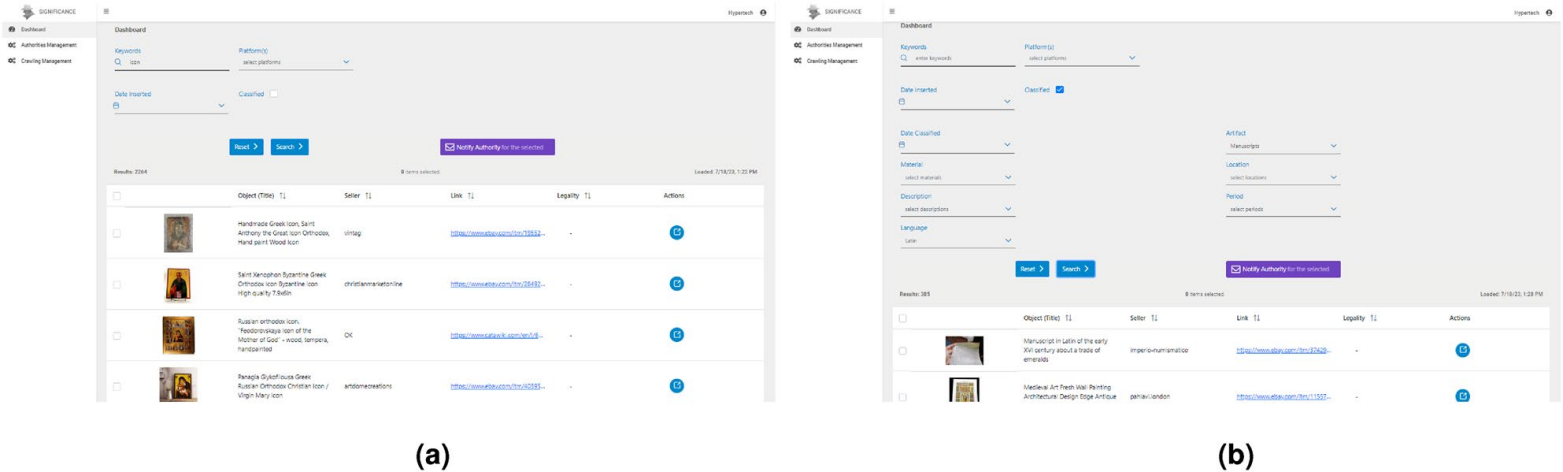
Examples of dashboard resulting from the previous experiments. (**a**) Selection of products through metadata collected by crawlers. (**b**) Selection of products through metadata obtained by AI.

**Figure 6:**
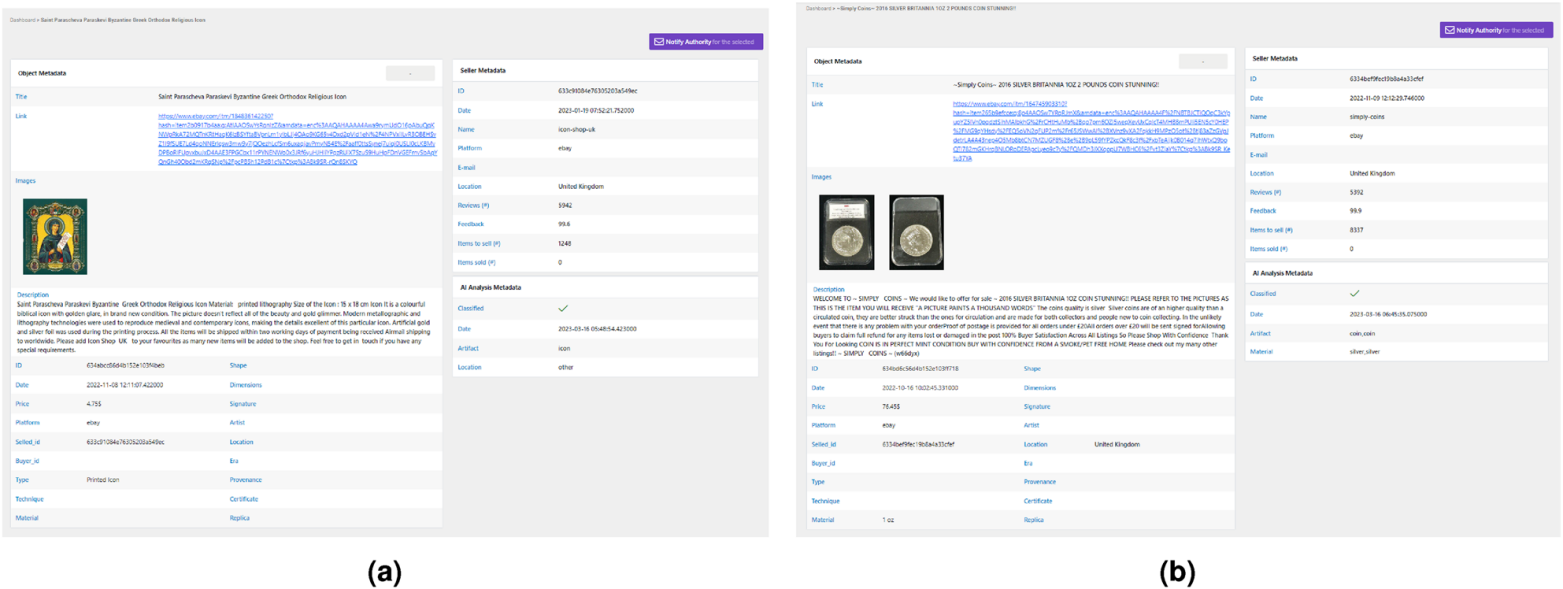
Examples of dashboard resulting from the previous experiments. Visualisation of the product and its metadata.

In this section, we present the results obtained from the implementation and evaluation of our SIGNIFICANCE Framework. We showcase the effectiveness of our approach in detecting and preventing the illicit movement of CH goods through comprehensive data analysis, DL and ontology-based classification, and real-time monitoring through our tailored platform. The results provide insights into the performance of our framework in accurately identifying and classifying artifacts, detecting suspicious activities, and triggering appropriate actions. We evaluate the performance of the AI module in terms of classification accuracy, precision, recall, and F1-score. Additionally, we assess the effectiveness of the ontology-based approach in enhancing the classification process and providing valuable contextual information. The classification task is performed to classify the goods and the fields of each artworks. In each experiment, the dataset was split between training and test set: 80% and 20%, respectively. The data were taken to maintain class balance in both datasets. We have decided not to make a validation set. The collected datasets are limited in terms of numerosity; as can be seen in Table 4, some classes have few samples. For this reason, we performed only a cursory analysis to check the feasibility of the approach, without hyperparameter optimization, so the validation set was not necessary. VGG16 network was pre-trained on Imagenet^42^. For each experiment, fine tuning was performed in mini-batch mode with batch size set to 32, using an adaptive optimization algorithm (Adam) with learning rate set to 0.001, to minimize a loss function defined as categorical cross entropy. The number of training epochs, set at 20, was established after preliminary experiments to avoid overfitting. Experiments were performed using 2 GPUs in parallel in the training phase. Only one GPU was used in the testing phase. Tables 3 and 4 report the data cardinality (number of images), for each class, used for the training and test of the models. The results are for both the phases defined. The results of the artworks classification (first classification phase) are reported in Table 5a. The following Tables reports the artworks features classification (second classification phase). In particular, Table 5b reports the results of Coins Material. Table 5c summarizes the results of Frescos Location classification; Table 5d reports the results of Frescos Period; Table 5e shows the results of the classification of Icons Location; Table 5f shows the results of Manuscripts Location; Table 5g reports the results of Manuscripts Language. Finally, Table 5h reports the results of Manuscripts Description. Furthermore, we present the outcomes of real-time monitoring through our tailored platform based on AI, showcasing the identification of unauthorized sales, illegal transfers, and trade of stolen CH artifacts across various online platforms. The effectiveness of the detection and prevention framework is evaluated by analyzing the number and nature of reported cases, as well as the timely actions taken to prevent further illicit movement. Moreover, we demonstrate the functionality and usability of the visualization platform developed as part of our framework. The interactive visualizations and dashboards provide a clear representation of the detected illicit activities, patterns, and insights derived from the collected data. The SIGNIFICANCE platform is the interface of the integrated SIGNIFICANCE system and provides a front-end application for visualization of collected data and automatic notification about identified illegal activities, as illustrated in Fig. 4. In more detail, it enables the user to search for specific CH object types (e.g. silver coin) found at specific online marketplaces and stores (e.g. eBay). It communicates with the Data Collection module and retrieves the associated collected data by calling the appropriate Rest services. By filling in a search form, it is possible to select keywords in the online product description, the shop of origin (Fig. 5a). In addition, for a search guided by the previously implemented AI module, it is possible to select labels (artworks classes and/or artworks features classes) obtained by processing product images (Fig. 5b). For each selected product, the page appears as in Fig. 6. These data are extracted from online sources using the Web Crawling components, while the AI modelling component uses them for updating the AI models and for automatic classification of post images. All the textual and visual information of the posts related to the item and the seller is depicted for assessment by the expert users (e.g., LEA authorities). The user can notify authorities of possible illegal online activities.

**Table 3:** Artworks classes.

Class	# Samples for class			# All samples		
	Total	Train	Test	Total	Train	Test
Coins	718	574	144	3590	2870	720
Frescoes	718	574	144			
Icons	718	574	144			
Manuscripts	718	574	144			
Others	718	574	144

**Table 4:** Artworks features.

Artefact	# Samples	Field	Class	# Samples for class			# All samples		
				Total	Train	Test	Total	Train	Test
Coins	2514	Material	Gold	838	671	167	2514	2013	501
			Silver	838	671	167			
			Other	838	671	167			
Frescoes	718	Location	Cyprus	248	198	50	718	574	144
			Italy	248	198	50			
			Other	222	178	44			
		Period	Byzantine	326	261	65	718	575	143
			Other	392	314	78			
Icons	1678	Locations	Cyprus	839	671	168	1678	1342	336
			other	839	671	168			
Manuscripts	> 366	Location	France	122	98	24	366	294	72
			Italy	122	98	24			
			Other	122	98	24			
		Language	Latin	161	129	32	322	258	64
			Other	161	129	32			
		Description	Figure	120	96	24	360	288	72
			Text	120	96	24			
			Text and figure	120	96	24

**Table 5:** Classification results.

	Precision	Recall	f1-score	Accuracy
(a) Artefact classification results				
Coins	0.99	1.00	1.00	0.97
Frescoes	0.94	0.95	0.94	
Icons	0.96	0.92	0.94	
Manuscripts	0.97	0.98	0.98	
Others	0.97	0.99	0.98	
Overall	0.97	0.97	0.97	
(b) Coins-material classification results				
Gold	0.99	0.98	0.98	0.92
Silver	0.93	0.82	0.87	
Others	0.85	0.96	0.90	
Overall	0.92	0.92	0.92	
(c) Frescoes-location classification results				
Cyprus	0.96	0.99	0.97	0.93
Italy	0.89	0.98	0.93	
Others	0.95	0.82	0.88	
Overall	0.93	0.93	0.93	
(d) Frescoes-period classification results				
Byzantine	0.94	0.92	0.93	0.94
Others	0.94	0.95	0.94	
Overall	0.94	0.94	0.94	
(e) Icons-location classification results				
Cyprus	0.98	0.99	0.99	0.99
Others	0.99	0.98	0.99	
Overall	0.99	0.99	0.99	
(f) Manuscripts-location classification results				
France	0.96	1.00	0.98	0.99
Italy	1.00	0.96	0.98	
Others	1.00	1.00	1.00	
Overall	0.99	0.99	0.99	
(g) Manuscripts-language classification results				
Latin	1.00	0.97	0.98	0.98
Others	0.97	1.00	0.98	
Overall	0.98	0.98	0.98	
(h) Manuscripts-description classification results				
Figure	0.88	0.96	0.92	0.93
Text	0.96	0.96	0.96	
Text and figure	0.95	0.88	0.91	
Overall	0.93	0.93	0.93	

### Limitations

The system developed so far makes it possible to isolate and identify objects retrieved from the web on online sales channels. AI algorithms/models trained on this dataset make it possible to automatically filter these types of objects. Currently, it is not possible to automatically classify whether an object is legal or illegal. However, through the developed platform, it is possible to filter objects by type, making it easier for experts to focus only on selected objects and indicate for each of them whether it is legal or illegal. To achieve the latter, it would be necessary to synthesise rules that can then be implemented by an algorithm (rule based); or, to annotate instances of legality or illegality in order to create a dataset from which to extract through statistical approaches, machine learning, deep learning, the function that connects the data that can be acquired with the status of legality/illegality. This requires the additional need to have experts such as archaeologists, art historians, cultural heritage experts etc in order to validate the results and not only to structure the databases for training. This is the only way to fully achieve the goal of early and automatic identification of illegal trade.

## Conclusions and future works

In this paper, we have presented the results and findings of our research on the detection and prevention of the illicit movement of CH goods. Through the implementation and evaluation of SIGNIFICANCE Framework, we have demonstrated its effectiveness in identifying and combating illicit activities in the online domain. The integration of comprehensive data collection, DL classification, ontology-based classification, and real-time monitoring has proven to be a powerful approach in detecting and preventing the illicit trade of CH artifacts. The deep learning model exhibited high accuracy in artifact classification, while the ontology-based approach enhanced the classification process by providing valuable contextual information. The visualization platform developed as part of our framework has contributed to a better understanding of the detected illicit activities, patterns, and insights derived from the collected data. It has empowered law enforcement agencies and relevant authorities with the necessary tools to make informed decisions, take proactive measures, and initiate targeted actions against illicit traffickers. Our research has provided significant contributions to the field of CH preservation and the prevention of illicit trafficking. By leveraging machine learning techniques, real-time monitoring, and interactive visualization, we have made significant strides in detecting and combating illicit activities in the online domain, safeguarding our CH. While our research has yielded promising results, there are several avenues for future work and improvement in the field of detecting and preventing the illicit movement of CH goods. Central to our future efforts is the optimisation of our deep learning model. We plan to harness the potential of transfer learning, ensemble methods and attention mechanisms to increase the accuracy and robustness of our artefact classification capabilities. This technical advancement will be complemented by a strategic expansion of our data sources through collaborations with a variety of institutions, organisations and law enforcement agencies. Such partnerships are essential not only to enrich our dataset, but also to gain a more accurate understanding of the complex patterns of illicit trafficking. In addition, we aim to establish a dynamic framework for continuous monitoring of online platforms, adapting our web-scraping algorithms and incorporating new technologies to effectively track and respond to evolving illicit activities. An overarching commitment to ethical research practices will guide all our future activities. We plan to rigorously address issues of privacy and data protection, ensuring that our methods are not only effective, but also ethically sound and compliant with international regulations and standards. The promotion of international co-operation is also a key element of our approach. By working with a range of global organisations, from CH institutions to law enforcement agencies, we aim to create a robust network for sharing insights, best practices and critical information. This collaboration is essential to developing comprehensive solutions to the global challenge of illicit trafficking in CH. Through these multifaceted efforts, we aim to significantly advance the field and create more effective tools and frameworks for preserving and protecting our CH for future generations. To address the limitation that the legal status of an artefact often depends on additional contextual information, such as provenance, ownership history, and legal documentation, which was not included in our dataset, future work will involve the creation of an ad hoc dataset containing comprehensive information for each artefact, incorporating data derived from stores and AI-extracted features. This dataset will be carefully labelled to distinguish between legal and illegal artefacts using the developed tool. By integrating this rich contextual information, we aim to improve the accuracy and reliability of the classification process.

## Data Availability

Data are available at: https://www.kaggle.com/datasets/afelix89/significance-dataset.
